# Effects of graded exercise rehabilitation on inflammatory factors and T-lymphocyte subsets in patients with acute exacerbation of chronic obstructive pulmonary disease: a randomized controlled trial

**DOI:** 10.3389/fmed.2025.1620577

**Published:** 2025-09-02

**Authors:** Yue Chen, Hong-Min Ran, Yan Wang, Dan-dan Fu, Na-na Yang, Chuan-li Cheng, Rong Liu, Lu-wen Luo, Ji-mei Luo, Li-na Ma, Hui Zeng

**Affiliations:** 1 The Second Affiliated Hospital of Zunyi Medical University, Zunyi, Guizhou, China; 2 Nursing School of Zunyi Medical University, Zunyi, Guizhou, China; 3 The Affiliated Hospital of Zunyi Medical University, Zunyi, Guizhou, China; 4 Department of Nursing, Zhejiang Cancer Hospital, Hangzhou, Zhejiang, China

**Keywords:** graded exercise rehabilitation, COPD exacerbation, immune function, inflammatory markers, T lymphocyte subsets

## Abstract

**Objectives:**

To evaluate the effects of an in-hospital graded exercise rehabilitation program on respiratory function, exercise capacity, inflammatory markers, and immune function in patients with acute exacerbation of chronic obstructive pulmonary disease (AECOPD).

**Methods:**

This is a prospective randomized controlled trial aimed at evaluating the efficacy of the graded exercise rehabilitation therapy based on the GOLD guidelines in patients with AECOPD. We divided the patients into the intervention group and the control group using the random number table method, with 35 patients in each group. The control group received conventional symptomatic treatment and exercise rehabilitation, while the intervention group underwent graded exercise rehabilitation according to the GOLD guidelines, twice a day, each session lasting 30–45 min, during hospitalization until discharge. After the treatment, the main indicators included serum inflammatory factors and T lymphocyte subsets. Secondary indicators included exercise endurance (6MWT), disease symptom burden (CAT), dyspnea (mMRC), self-care ability (ADL), and psychological state (HADS). The hospitalization time, duration of non-invasive mechanical ventilation, and the overall incidence of related complications were also evaluated. All evaluations were conducted at baseline and at the end of the in-hospital rehabilitation intervention before discharge.

**Results:**

Baseline characteristics were comparable between groups. Post-intervention, both groups showed significant improvements in all measured parameters (*p* < 0.05 vs. baseline), with superior outcomes in the intervention group: greater reductions in inflammatory markers (IL-6, IL-8, TNF-*α*, CRP; *p* < 0.05); more favorable immune profile (higher CD3^+^, CD4^+^, CD4^+^/CD8^+^ ratio; lower CD8^+^; *p* < 0.05); better functional outcomes (6MWT, mMRC, CAT, ADL, HADS; *p* < 0.05). The duration of non-invasive ventilation, the length of hospital stay, and the incidence of complications were all reduced.

**Conclusion:**

The GOLD-based graded exercise rehabilitation demonstrates superior clinical efficacy compared to conventional rehabilitation for AECOPD patients, showing significant benefits in reducing systemic inflammation, improving immune function, and enhancing physical and psychological outcomes.

## Introduction

Chronic obstructive pulmonary disease (COPD) is a chronic respiratory condition characterized by persistent airflow limitation, featuring progressive airflow obstruction and abnormal inflammatory responses of the lungs to noxious particles or gases (1, 2). Globally, COPD remains a leading cause of morbidity and mortality, ranking as the third most common cause of death worldwide according to WHO statistics, surpassed only by ischemic heart disease and stroke (3).

Acute exacerbations of COPD (AECOPD) are defined as acute worsening of respiratory symptoms including dyspnea, cough, and sputum production (4, 5). These exacerbations not only accelerate lung function decline but also increase risks of respiratory failure and mortality (6). Elderly AECOPD patients are particularly vulnerable due to age-related physiological decline and compromised immune function (7), necessitating special consideration during acute-phase management. However, current research has predominantly focused on pulmonary rehabilitation in stable COPD, with relatively limited evidence regarding rehabilitation during acute exacerbations.

As the cornerstone of pulmonary rehabilitation, exercise training has been proven to effectively improve patients’ respiratory function and exercise endurance (8), while reducing systemic inflammation and regulating immune responses (9). Meanwhile, studies have shown that early pulmonary rehabilitation initiated during hospitalization for AECOPD patients is beneficial, improving their quality of life, activity capacity, and reducing readmission rates and mortality (10–12). The therapeutic effect is influenced by multiple factors, including exercise mode, intensity, duration, and environment. The best outcome requires a precisely tailored and safely implemented program. Current rehabilitation programs mainly focus on exercise mode, timing, safety, and effectiveness, often neglecting the critical aspect of stratifying the severity of acute exacerbation. Based on our preliminary research, we have developed a personalized and graded exercise rehabilitation program according to the 2023 GOLD guideline classification system (13), categorizing the severity of AECOPD into three levels based on clinical manifestations and arterial blood gas analysis, thereby achieving truly dynamic and precise exercise therapy.

Emerging evidence highlights the prognostic value of inflammatory cytokines and T-cell subsets in predicting AECOPD recurrence and outcomes (14). To address current knowledge gaps, we conducted a randomized controlled trial to systematically evaluate the therapeutic effects of graded exercise rehabilitation in AECOPD patients, with particular focus on its impacts on inflammatory markers and T-lymphocyte subpopulations. This investigation aims to provide novel theoretical foundations and practical approaches for AECOPD rehabilitation, ultimately optimizing clinical management strategies and improving patients’ quality of life and long-term prognosis.

## Materials and methods

### Study design

This is a prospective, single-blind, randomized controlled clinical trial conducted in accordance with the CONSORT guidelines and the Helsinki Declaration. It was implemented in Guizhou, China from August 2024 to February 2025. The study has been registered in the Chinese Clinical Trial Registry (Registration Number: ChiCTR2300072409). The study has received ethical approval from the Medical Ethics Committee of the Second Affiliated Hospital of Zunyi Medical University (approval number: 2024-1-295). All participants and their families were informed of the study and signed informed consent forms.

### Participants

The study population comprised patients aged 40–80 years with a confirmed COPD diagnosis per 2023 GOLD criteria, who were hospitalized for acute exacerbation (AECOPD). Eligible participants met the following criteria: (1) clear consciousness with unimpaired verbal communication; (2) no prior participation in structured exercise training programs.

Exclusion criteria: Severe cardiovascular diseases, acute exacerbation of bronchial asthma, pulmonary embolism, pneumothorax, hemoptysis, metastatic cancer, diseases affecting neuromuscular system or bone and joint disorders, limb impairments, deep vein thrombosis, immunodeficiency or low immunity patients, as well as those with poor compliance and unwillingness to cooperate.

### Randomization and allocation

At baseline assessment, an independent researcher not involved in either evaluation or intervention procedures allocated participants using a computer-generated randomization table. This staff member prepared the randomization sequence in advance and assigned consecutive eligible patients according to their admission order—those corresponding to odd numbers were allocated to the control group while even numbers designated the intervention group.

### Interventions

After completing the registration, eligible participants need to undergo a baseline assessment. After a rigorous evaluation, if the patient’s condition and vital signs were stable, exercise rehabilitation training was initiated within 24 h after admission and continued during hospitalization until discharge. Before each training session, vital signs and consciousness status will be evaluated.

Both groups of patients will receive the standard treatment protocol, which covers multiple aspects: For the use of antibiotics, based on the patient’s clinical symptoms, signs, and results of sputum culture and other examinations, targeted antibiotics are selected for anti-infection treatment. The treatment course is adjusted according to the severity of the condition and the treatment effect; The use of systemic corticosteroids follows relevant guidelines, and appropriate doses and treatment courses of oral or intravenous administration are given based on the patient’s condition to reduce airway inflammation. At the same time, conventional drugs such as bronchodilators, expectorants, etc. are also combined for comprehensive treatment, as well as necessary oxygen therapy and respiratory support measures.

#### Control group

Received standard rehabilitation comprising.

##### Pursed-lip breathing

Nasal inhalation followed by prolonged expiration through pursed lips (expiratory duration ≈2 × inspiratory time), administered twice daily (5–10 min/session).

##### Diaphragmatic breathing

Pursed-lip breathing: Performed in supine/sitting position with chest immobilization (abdominal retraction during inspiration/relaxation during expiration) at identical frequency/duration.

##### ADL training

Pursed-lip breathing: Self-care activities (feeding, dressing) and basic limb exercises (hand gripping, elbow flexion). Ambulatory patients progressed to walking (2 × daily, 8–12 reps/set, 2–3 sets).

#### Intervention group

Received graded exercise rehabilitation training. The grading standard for the severity of AECOPD patients hospitalized is shown in Table 1. The specific plan is shown in Table 2.

**Table 1:** Grading criteria for severity of AECOPD inpatients.

Classification	Basis
Level I	RR ≤ 24 times/min; HR < 95 times/min; No application of accessory respiratory muscles; no altered mental state; Hypoxemia can be improved by inhalation of 24–35% oxygen concentration; No PaCO_2_ increase
Level II	R > 24 beats /min; Application of auxiliary respiratory muscle group; No altered mental state of consciousness; Improved hypoxemia by inhaling >35% oxygen concentration; Hypercapnia is an increase in PaCO_2_ from baseline or to 50–60 mmHg
Level III	R > 24 beats /min; Application of auxiliary respiratory muscle group; Sharp changes in mental state of consciousness and can cooperate with treatment; Need to inhale >40% oxygen concentration to improve hypoxemia; Hypercapnia, an increase in PaCO_2_ > 60 mmHg from baseline or acidosis (PH ≤ 7.25)

**Table 2:** Graded exercise rehabilitation in patients with AECOPD.

Exercise grading	Evaluation criteria	Sports content
Level I Sports	Level III patients: respiratory rate >24 times/min; application of auxiliary respiratory muscles; The need for inhaled >40% oxygen concentration improves hypoxemia; Hypercapnia is defined as an increase in PaCO_2_ > 60 mmHg or acidosis (pH ≤ 7.25).	1. Respiratory Training: Instruct patients to close their mouths and practice regular, relaxed breathing to achieve synchronization with the ventilator and enhance the effectiveness of ventilator therapy. 2. Exercise training: mainly passive exercise, and gradually transition to active exercise according to the patient’s condition. (1) Multi-channel sensorimotor stimulation: massage and patting of the limbs to strengthen sensory afferent. (2) Upper limb training. ① Joint relaxation: flexion and extension of wrist, elbow and shoulder joints of both upper limbs. ② Fist clenching exercise: clench your fist firmly for 5 s, and then release it for 5 s. ③ Arm raising exercise: Assist the patient to take the supine or sitting position, put the hands flat on the sides of the body, and raise the upper limbs flat at 180°. (3) Lower limb training. ① Joint relaxation: flex the hip and knee joints of both lower limbs at 90°, and exercise alternately with both legs. ② Straight leg raising exercise: keep the knee joint straight on one side of the limb, raise the heel about 15 cm from the bed surface with dorsal flexion of the foot, and alternate the legs. ③ Ankle pump exercise: the patient’s ankle joint is toe flexion, dorsal flexion and circumambulation, all of which are maintained for 5 ~ 10 s, exhaling when exerting force and inhaling when relaxing. Complete 2 ~ 3 sets/times, 8 ~ 12 times/group for each action.
Level II Sports	Level II patients: respiratory rate >24 times/min; application of auxiliary respiratory muscles; Hypoxemia can be improved by inhalation of >35% oxygen concentration; Hypercapnia, that is, PaCO_2_ rises to 50 ~ 60 mmHg.	1. Respiratory muscle training (1) Pursed lip abdominal breathing: take a sitting or lying position, place your hands on the chest and abdomen respectively, expand the abdomen outward to the maximum extent when inhaling through the nose, so that the abdomen is bulging, and the chest remains still, when exhaling, the pursed lips are slightly closed and the abdomen is naturally concave, and the abdomen is contracted in the direction of the spine to the maximum extent, and the chest remains still, and the ratio of inhalation and exhalation is 1:2 or 1:3. 5 ~ 10 min/time. 2. Athletic training (1) Upper limb training. ① Elbow extension exercises: take a flat lying position, straighten the elbow joint, and then flex the elbow joint, so that the hands are as close to the shoulder joint as possible, and the hands are alternately practiced. ② Stretch sit-ups: Hold the bed rails with both hands, use the strength of the upper limb muscles to sit up slowly, exhale when you get up hard, and inhale slowly while lying down. ③ Grip strength exercises: use grip strength devices, fists with hands, etc. to train forearm muscles, pectoralis major muscles, biceps brachii and triceps brachii muscle groups. (2) Lower limb training. ① Straight leg raising exercise: take the supine position, straighten the knee joint, raise the heel about 15 cm from the bed surface with dorsal flexion of the foot, exhale when lifting the leg hard, inhale when slowly lowering, and exercise alternately with both legs. ② Bridge exercise: take the supine position, the knee joint is flexed, the soles of the feet are flat on the bed, and the buttocks are lifted 10 ~ 15 cm away from the bed surface, and then slowly put down for 5 ~ 10 s as much as possible. ③ Cycling in the air: Take a flat lying position, keep the upper body still, bend the knees and raise the legs, and alternate the two calves in the air to pedal the bicycle. Complete 2 ~ 3 sets/times, 8 ~ 12 times/group for each action. 3. Transfer training: After the patient’s condition improves, assist the patient to get out of bed and hold the bed rails to stand at the foot of the bed or beside the bed, step in place, and move at the bedside.
Level III Sports	Level I patients: respiratory rate ≤24 breaths/min; Heart rate <95 beats/min; No application of accessory respiratory muscles; Hypoxemia can be improved by inhaling 24 ~ 35% oxygen concentration; No PaCO_2_ increase.	1. Respiratory muscle resistance training (1) Pursed lip abdominal resistance training: take the supine position, place a sandbag on the abdomen on the basis of pursed lip abdominal breathing for abdominal muscle resistance training, and gradually increase the weight of the sandbag from 0.5 kg to 2 kg according to the patient’s tolerance, 3 ~ 5 min/time. (2) Artificial resistance breathing training: take balloon blowing and other methods, hold your breath slightly after deep inhalation, and then blow hard to inflate the balloon, reduce the residual air volume in the lungs, maintain a certain pressure in the trachea, and prevent premature deflation of the bronchi and small bronchi, 3 ~ 5 min/time. 2. Athletic resistance training (1) Upper limb resistance training: open your feet shoulder-width apart, lift sandbags with both hands or tie sandbags with your wrists to front/side raises, flex and extend your arms at the back of your neck, and curl your arms to train your upper limb muscles. (2) Resistance training of lower limbs: stand firmly on a chair or wall, keep the body neutral, tighten the abdomen and buttocks, straighten the knee joint, tie sandbags to the lower legs, and do exercises such as extension, abduction and knee flexion of the thighs; Sit in a chair and straighten and flex your calves. The weight of the sandbag was gradually increased from 0.5 kg to 2 kg according to the patient’s tolerance. Complete 2 ~ 3 sets/times, 8 ~ 12 times/group for each action. 3. Aerobic exercise (1) Walking training: Start with the 40% intensity walking distance of the 6-min walking test, and increase the distance by 10% when the Borg score decreases by 1 point from baseline. (2) Stair climbing training: up and down stair training, climb 25 stairs for the first time, and add 5 stairs each time if the patient tolerates.

### Outcome measurement

#### Primary observation measures

##### Measurement of inflammatory markers

Fasting venous blood samples (3 mL) were collected from the antecubital vein pre- and post-intervention. Serum was isolated by centrifugation at 3000 rpm for 10 min. IL-6, IL-8, TNF-*α*, and hs-CRP levels were quantified using ELISA kits (Shanghai Jingkang Biological Engineering Co., Ltd.) with a Sunrise automated microplate reader (Tecan, Switzerland) via sandwich immunoassay. All procedures strictly adhered to manufacturer protocols and were performed by certified technicians.

##### Immunological function assessment

Fasting venous blood (5 mL) was collected in heparinized tubes and mixed thoroughly. T lymphocyte subsets (CD3^+^, CD4^+^, CD8^+^) and CD4^+^/CD8^+^ ratios were analyzed using a BriCyte E6 flow cytometer (Mindray, Shenzhen) with matched antibody kits (Mindray Bio-Medical Electronics Co., Ltd.). Standardized protocols were followed for.

### Secondary observation measures

#### Exercise capacity

The 6-min walk test (6MWT) was performed along a 30-meter corridor, with continuous monitoring of walking distance, oxygen saturation (SpO_2_), heart rate, and exercise-induced symptoms.

#### Dyspnea severity

Assessed using the modified Medical Research Council (mMRC) (15) dyspnea scale (range: 0–4), where higher scores indicate greater dyspnea limitation.

#### Health status

The health status assessment was conducted using the Chronic Obstructive Pulmonary Disease Assessment Test (CAT) (16), with a score range of 0–40. This test mainly reflects the burden of symptoms for the patients, and the lower the score, the less the patient is affected by the disease.

#### Functional status

Activities of daily living (ADL) (17) were evaluated using the Barthel Index (range: 0–100), where higher scores denote greater independence.

#### Psychological assessment

The Hospital Anxiety and Depression Scale (HADS) (18) (range: 0–21 per subscale) evaluated. Anxiety (scores ≥8 suggestive, ≥11 diagnostic). Depression (scores ≥8 suggestive, ≥11 diagnostic).

#### Clinical outcomes

Duration of non-invasive ventilation (days), length of hospitalization (days), incidence of complications (%).

### Statistical analysis

The data analysis was conducted by an independent statistician who was unaware of the grouping situation using SPSS 29.0. Continuous variables were expressed as mean ± standard deviation or median (quartile), and within-group comparisons were performed using paired t-test, while between-group comparisons were conducted using independent sample t-test; categorical variables were presented as number (percentage), and chi-square test was used for analysis. A difference was considered statistically significant when *p* < 0.05 (two-sided).

## Results

**Figure 1:**
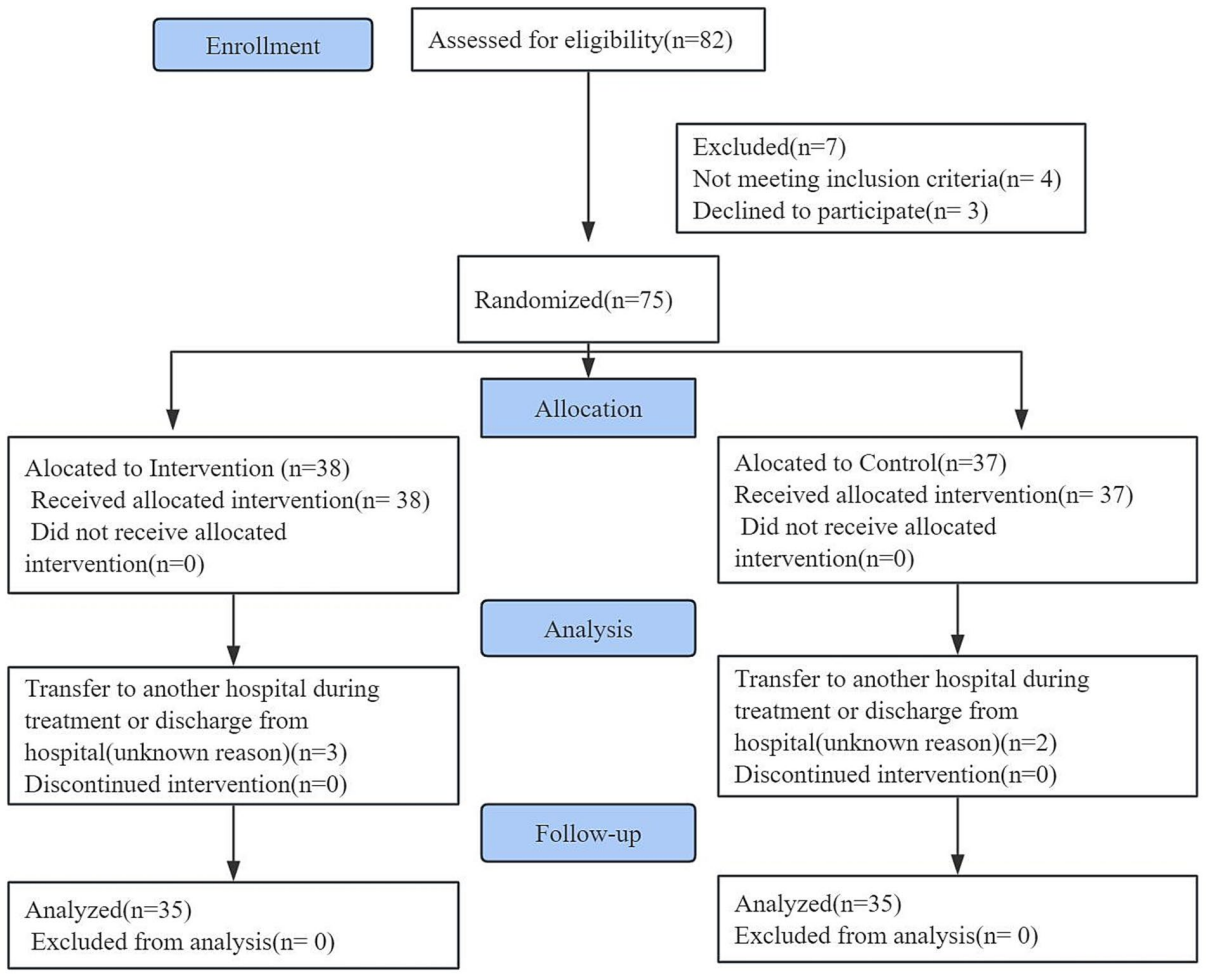
Flow diagram.

From August 2024 to February 2025, 82 patients were initially screened. After excluding 4 ineligible patients and 3 decliners, 75 participants were randomized to either the intervention (*n* = 38) or control group (*n* = 37). Five cases were subsequently lost to follow-up due to hospital transfer or discharge, yielding 35 analyzable cases per group (Figure 1). Baseline characteristics demonstrated balanced distribution between groups (*p* > 0.05, Table 3).

**Table 3:** Comparison of general data between the two groups.

Characteristic	Intervention group (*n* = 35)	Control group (*n* = 35)	Statistics	*p*-value
Age, years [mean (standard deviation)]	68.69 ± 8.55	65.46 ± 9.06	1.533	0.130
Gender, *n* (%)			0.025	0.771
Male	28 (80.0)	27 (77.1)		
Female	7 (20.0)	8 (22.9)		
Body mass index (BMI) [mean (standard deviation)]	22.43 ± 4.91	23.03 ± 3.16	−0.604	0.548
Duration of disease, years [mean (standard deviation)]	7.57 ± 6.81	9.55 ± 12.10	−0.842	0.402
AECOPD level, *n* (%)			0.031	0.850
Level I	14 (40.0)	15 (42.9)		
Level II	12 (34.3)	13 (37.1)		
Level III	9 (25.7)	7 (20.0)		
CAT, score [mean (standard deviation)]	26.74 ± 4.79	27.40 ± 3.77	−0.622	0.536
mMRC, score [mean (standard deviation)]	3.11 ± 0.83	2.86 ± 0.77	1.340	0.185
Pulmonary function [mean (standard deviation)]				
FEV_1_, L	1.22 ± 0.62	1.11 ± 0.41	1.484	0.143
FVC, L	1.68 ± 0.48	1.52 ± 0.45	1.489	0.142
FVC/Pre, %	52.15 ± 14.25	53.22 ± 16.89	0.246	0.807
FEV_1_/Pre, %	49.87 ± 20.12	48.56 ± 19.37	0.387	0.699
Number of acute exacerbations in the past year, times [M (P25, P75)]	2.00 (1.00, 2.00)	1.00 (1.00, 2.00)	−1.087	0.277
Occupation, *n* (%)			0.633	0.546
Workers and peasants	28 (80.0)	30 (85.7)		
Civilian personnel	2 (5.7)	2 (5.7)		
Freelancer	5 (14.3)	3 (8.6)		
Educational level, *n* (%)			0.117	0.209
Elementary school and below	23 (65.7)	23 (65.7)		
Junior high school	8 (22.9)	10 (28.6)		
Technical secondary school or above	4 (11.4)	2 (5.7)		
Place of residence, *n* (%)			0.093	0.387
Town	10 (28.6)	12 (34.3)		
Countryside	25 (71.4)	23 (65.7)		
Spouse situation, *n* (%)			0.448	0.337
Married	26 (74.3)	30 (85.7)		
Unmarried	2 (5.7)	0 (0)		
Divorce	1 (2.9)	0 (0)		
Widowed	6 (17.1)	5 (14.3)		

### The main observation results are as follows

**Figure 2:**
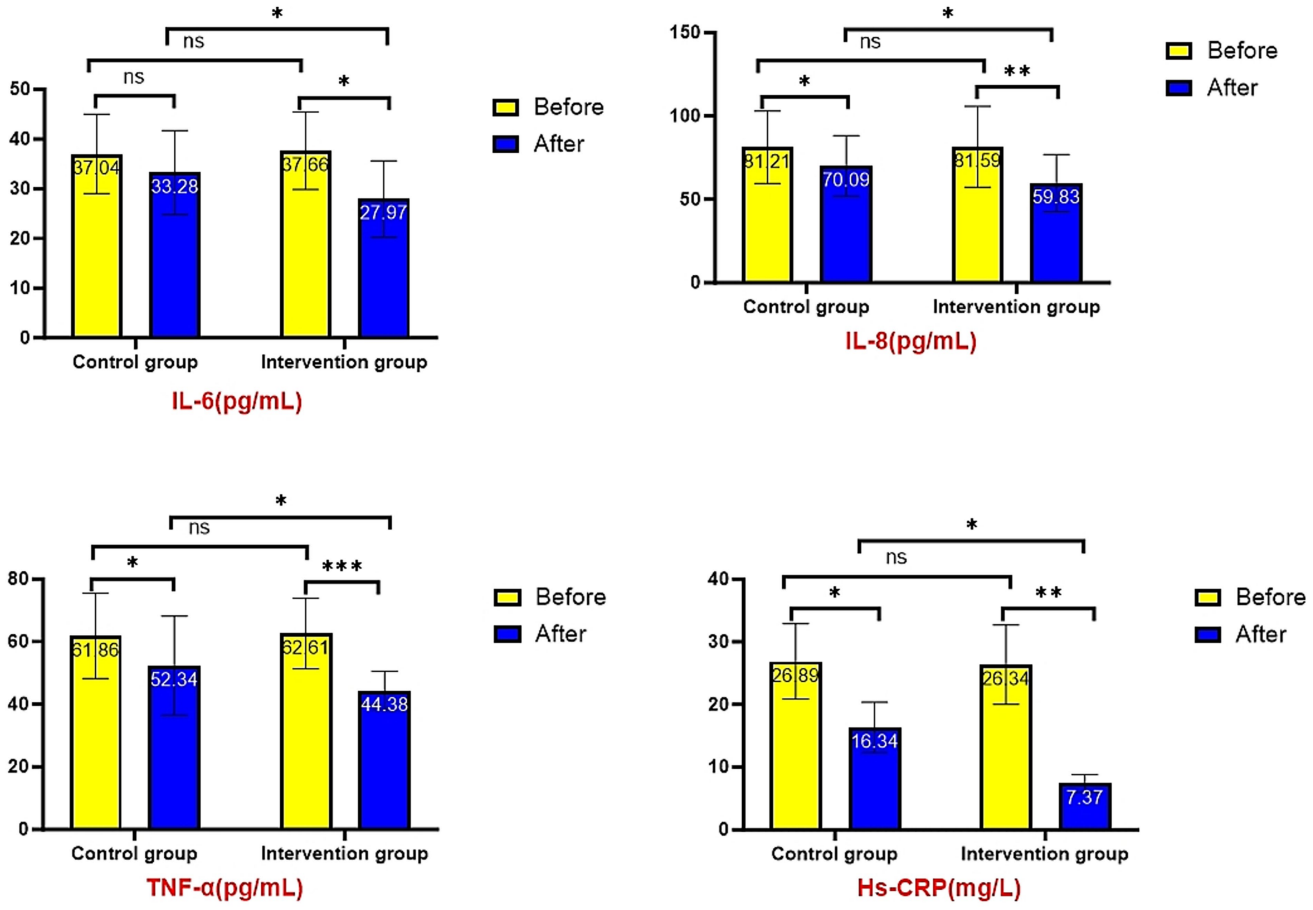
Comparison of serum inflammatory factor levels.

Serum inflammatory factor analysis revealed no significant intergroup differences at baseline (all *p* > 0.05). Post-intervention results demonstrated significantly lower levels of IL-6 (27.97 ± 7.64 vs. 33.28 ± 8.44 pg./mL, *p* = 0.012), IL-8 (59.83 ± 17.09 vs. 70.09 ± 18.12 pg./mL, *p* = 0.026), TNF-*α* (44.38 ± 6.14 vs. 52.34 ± 15.83 pg./mL, *p* = 0.016), and hs-CRP (7.37 ± 2.15 vs. 15.29 ± 4.62 mg/L, *p* = 0.042) in the intervention group compared to controls. Both groups showed significant improvements from baseline (*p* < 0.05), with greater reductions observed in the intervention group (Figure 2). (NS: not significant; *p < 0.05; **p < 0.01; ***p < 0.001. All the following expressions are like this.)

**Figure 3:**
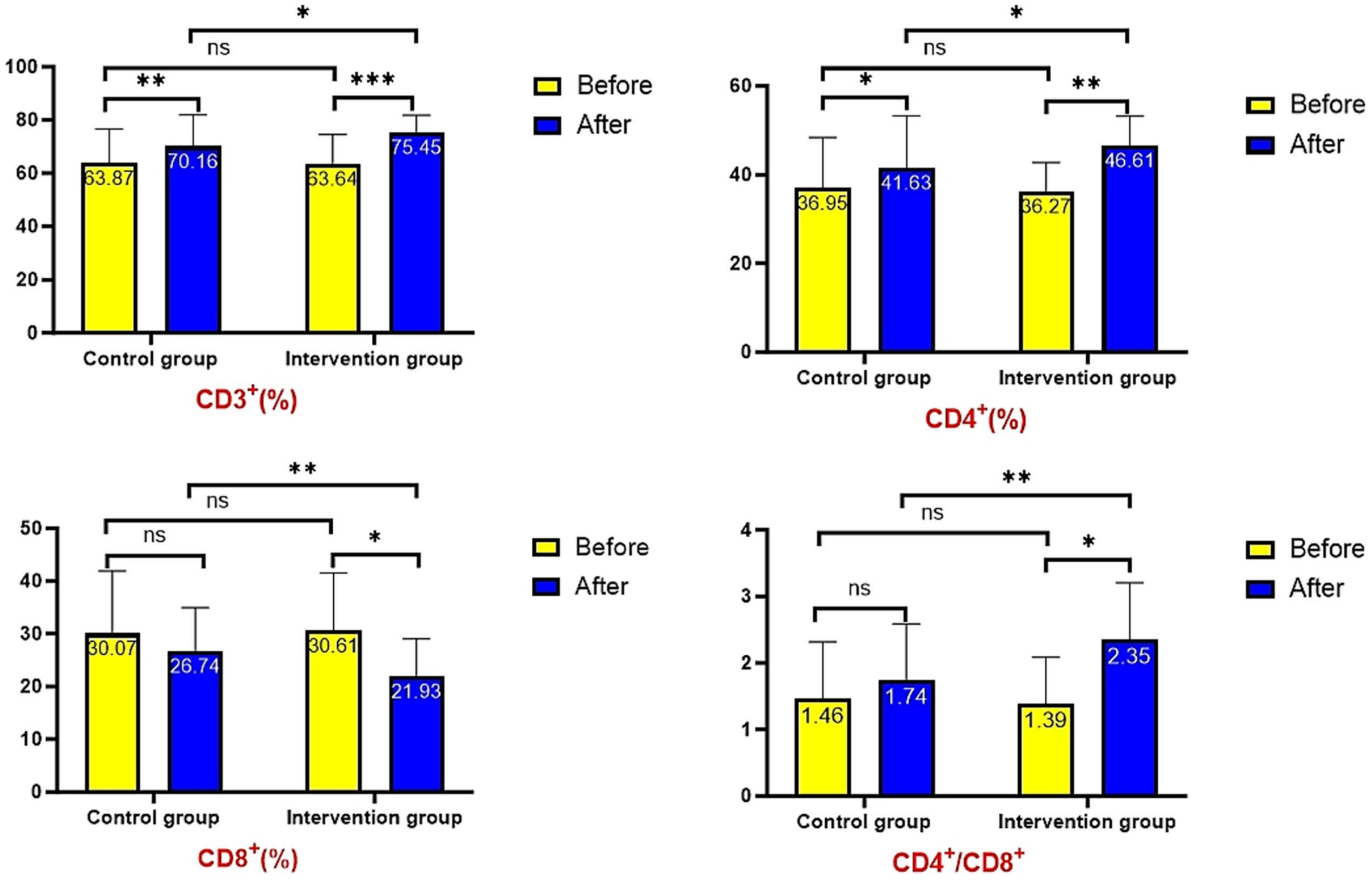
Comparison of immune function indicators.

Baseline measurements showed no significant differences in T lymphocyte subsets (CD3^+^, CD4^+^, CD8^+^) or CD4^+^/CD8^+^ ratio between groups (*p* > 0.05). Post-intervention, the intervention group demonstrated significantly higher CD3^+^ (75.45 ± 6.37% vs. 70.16 ± 11.73%, *p* = 0.023), CD4^+^ (46.61 ± 6.57% vs. 41.63 ± 11.58%, *p* = 0.031), and CD4^+^/CD8^+^ ratio (2.35 ± 0.86 vs. 1.74 ± 0.85, *p* = 0.004), along with significantly lower CD8^+^ levels (21.93 ± 7.09% vs. 26.74 ± 8.18%, *p* = 0.011) compared to controls. Longitudinal analysis revealed significant increases in CD3^+^ and CD4^+^ levels from baseline in both groups (*p* < 0.05), while changes in CD8^+^ levels and CD4^+^/CD8^+^ ratio remained non-significant (*p* > 0.05, Figure 3).

### The results of secondary observation indicators are as follows

**Figure 4:**
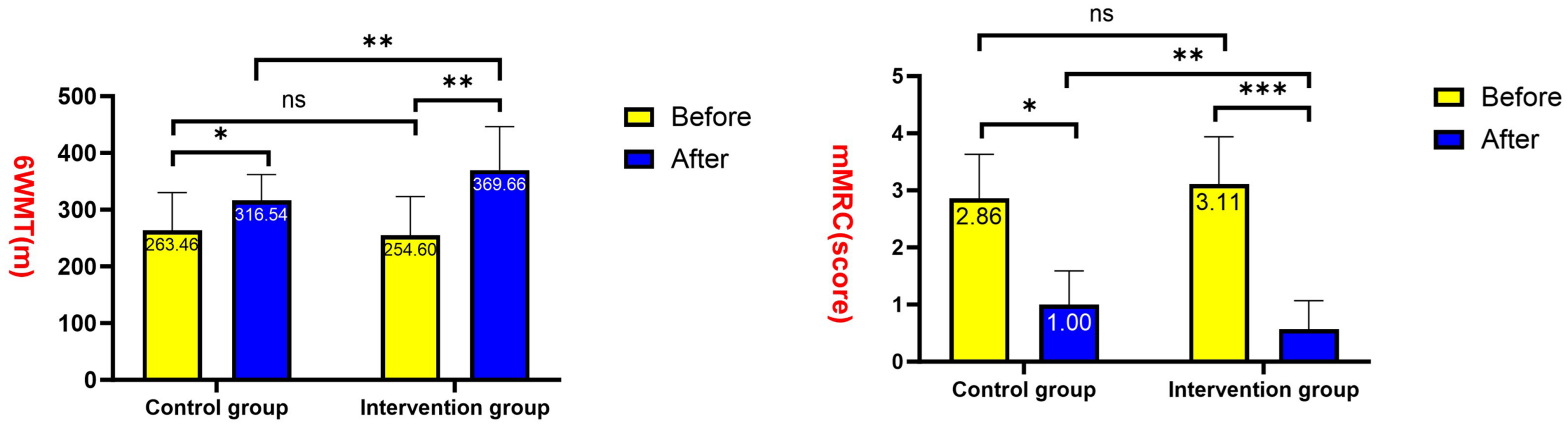
Comparison of 6MWT (meters) and mMRC (score). *p < 0.05, **p < 0.01, and ***p < 0.001.

Exercise Capacity and Respiratory Function Outcomes: Post-intervention analysis revealed significantly better performance in the intervention group compared to controls for both 6-min walk test distance (369.66 ± 76.69 m vs. 316.54 ± 45.4 m, *p* = 0.001) and modified Medical Research Council (mMRC) dyspnea score (1.57 ± 0.5 vs. 2.00 ± 0.59, *p* = 0.002). Baseline measurements showed no significant differences between groups for either parameter (*p* > 0.05). Following treatment, both groups demonstrated significant improvements from baseline in 6WMT distance (*p* < 0.05) and mMRC scores (both *p* < 0.05), with the intervention group exhibiting greater magnitude of improvement (Figure 4).

**Figure 5:**
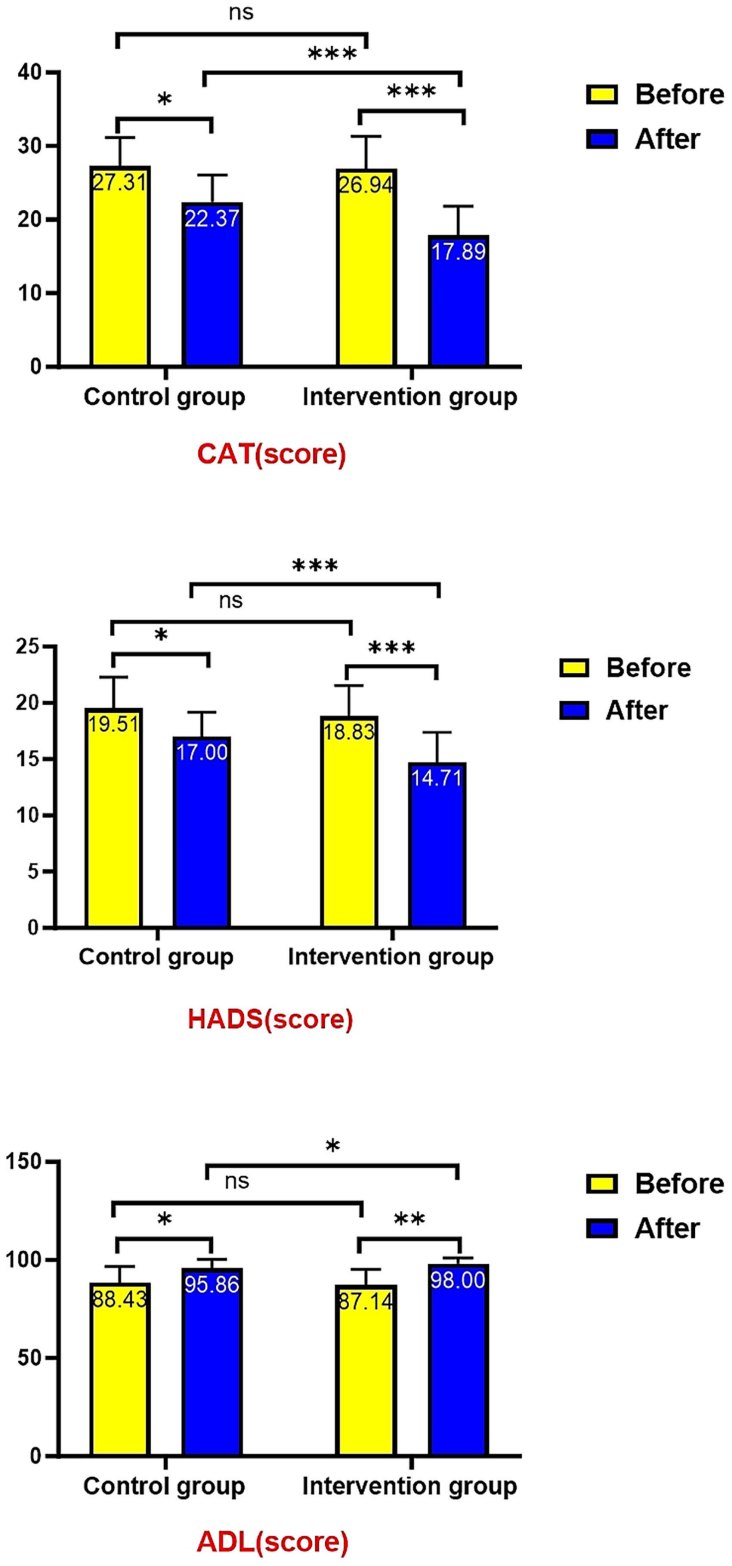
Comparison of CAT, HADS and ADL Scores.

Post-treatment intergroup comparisons demonstrated significantly better outcomes in the intervention group across multiple key indicators: CAT score (17.89 ± 3.92 vs. 22.37 ± 3.68, *p* < 0.001), HADS score (14.71 ± 2.66 vs. 17.00 ± 2.17, *p* < 0.001), and ADL score (98.00 ± 3.02 vs. 95.86 ± 4.62, *p* = 0.025). Baseline measurements showed no significant differences between groups in CAT scores, HADS scores, or ADL scores (all *p* > 0.05). Following treatment, both groups exhibited significant improvements compared to baseline: CAT and HADS scores decreased significantly (both *p* < 0.05) while ADL scores increased significantly (both *p* < 0.05), with the intervention group showing more pronounced improvements (Figure 5).

**Figure 6:**
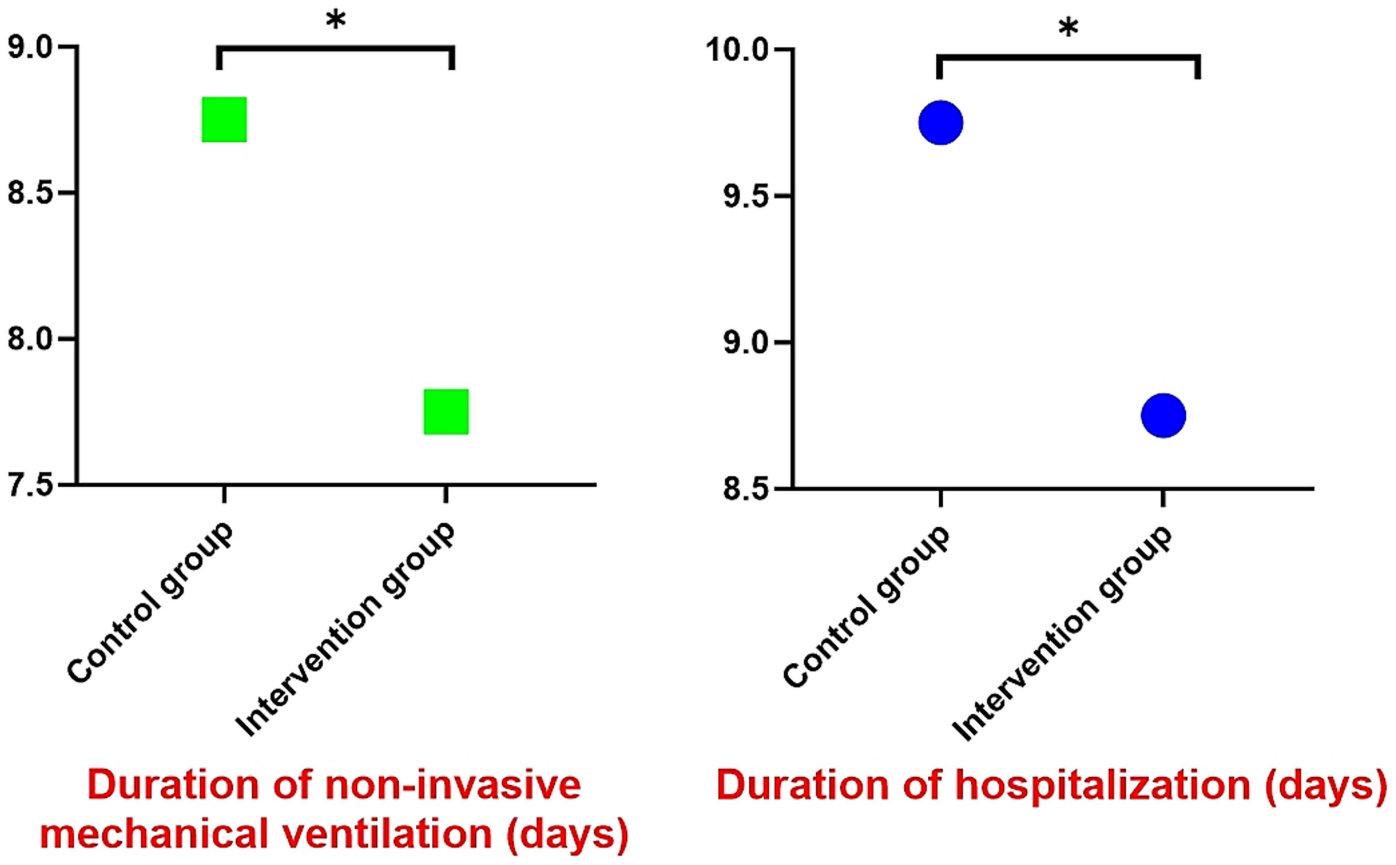
The duration of non-invasive mechanical ventilation and the length of hospital stay.

The intervention group demonstrated significantly shorter durations of both non-invasive mechanical ventilation [7.75 (6.75–9.75) days vs. 9.75 (7.75–11.25) days, *p* = 0.034] and hospitalization [8.75 (7.50–10.00) days vs. 9.75 (8.50–11.75) days, *p* = 0.035] compared to the control group, with median differences of 2 days and 1 day respectively, showing statistically significant improvements (Figure 6).

**Figure 7:**
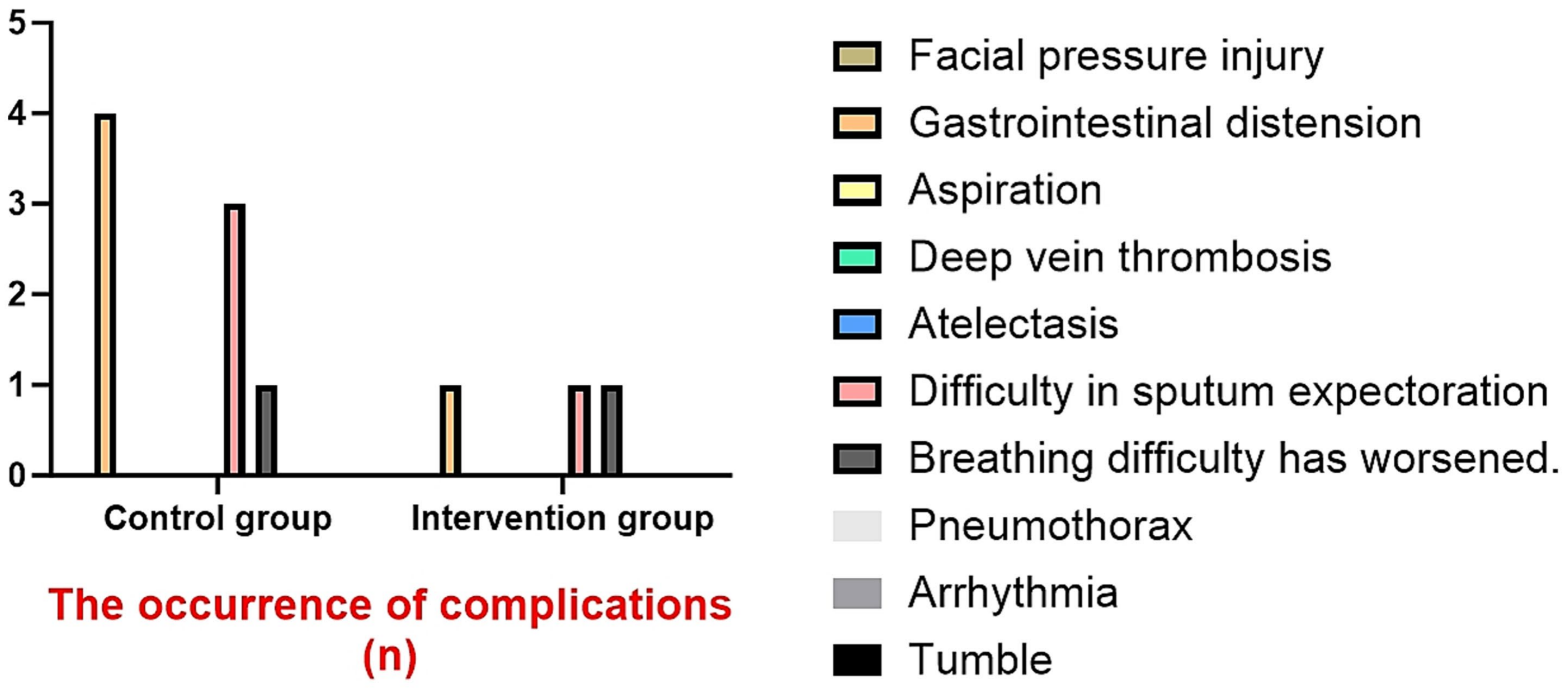
The occurrence of complications.

The incidence of complications in the intervention group was lower than that in the control group. The incidence of gastrointestinal distension was 2.86% versus 11.43%, while the incidence of difficulty in expectoration was 2.86% versus 8.57%. The cases of exacerbated breathing difficulties in the intervention group were the same as those in the control group, both being 2.86%. It is worth noting that neither group experienced severe complications (such as aspiration, deep vein thrombosis, pneumothorax, etc.). Arrhythmias and falls were also rare, with only 1 case (2.86%) of fall in the control group. These results support the safety of early rehabilitation in this population (Figure 7).

## Discussion

COPD is a highly prevalent global respiratory disorder, has emerged as one of the leading chronic conditions threatening human health. The disease not only causes progressive decline in lung function with symptoms including dyspnea, cough, and sputum production, but also severely restricts patients’ daily activities and social participation, imposing substantial economic and psychological burdens on families and society (19). During acute exacerbations (AECOPD), clinical deterioration accelerates pulmonary function impairment and may trigger life-threatening complications such as cor pulmonale and respiratory failure (20, 21). Epidemiological data indicate COPD patients experience 0.5–3.5 acute exacerbations annually, with increased frequency exacerbating healthcare burdens and significantly elevating mortality risk (22). Current pharmacological interventions demonstrate limited efficacy, while neglecting rehabilitation often leads to disease recurrence and further quality of life deterioration (23). The predominance of elderly individuals among AECOPD patients—who face compounded challenges from both physiological decline and immune dysfunction—renders their treatment and rehabilitation particularly demanding (24, 25). Our preliminary research and existing literature (13, 26) have confirmed that exercise rehabilitation significantly improves exercise tolerance, alleviates dyspnea, and enhances quality of life in AECOPD patients. Building upon this foundation, the present study specifically investigates the effects of graded exercise rehabilitation on immune function and inflammatory cytokine profiles in AECOPD patients.

The pathogenesis of AECOPD involves complex interactions, with most researchers attributing it to immune dysfunction mediated by T lymphocytes and their associated chemokine-induced inflammatory responses (27). Persistent airway inflammation in AECOPD patients is characterized by markedly elevated levels of inflammatory cytokines (IL-6, IL-8, TNF-*α*), which not only reflect disease severity but also perpetuate chronic inflammatory damage to airways, lung parenchyma, and vasculature through neutrophil activation (28). A prospective study of 1,755 patients demonstrated significantly higher serum levels of TNF-*α*, IL-6, and CRP in COPD patients compared to healthy controls, with longitudinal data revealing strong correlations between these inflammatory markers and frequency of exacerbations, degree of pulmonary function impairment, and mortality rates (29). Our results align with these established findings: both groups showed significant post-treatment reductions in serum IL-6, IL-8, TNF-α, and CRP levels compared to baseline (*p* < 0.05), with the intervention group demonstrating superior reductions versus controls (*p* < 0.05). These observations corroborate previous work by Dou Yu, Gaunaurd, and Fischer (30–32), collectively indicating that graded exercise rehabilitation effectively mitigates systemic inflammation in AECOPD patients.

Existing evidence indicates that AECOPD patients exhibit reduced cellular immunity, with T lymphocytes playing pivotal roles as both immunoregulators and effector cells (33). Among T-cell subsets, CD8^+^ cells recognize antigens and mediate cytotoxicity against altered host cells, while CD4^+^ helper T cells (B-cell inducing subset) express CD4^+^ antigens (34). A decreased CD4^+^/CD8^+^ ratio reflects impaired immune competence and elevated infection risk, establishing the clinical relevance of T-lymphocyte subset alterations in COPD (35, 36). Our results demonstrate that graded exercise rehabilitation significantly increased CD3^+^, CD4^+^ percentages and CD4^+^/CD8^+^ ratios while reducing CD8^+^ levels in both groups, with more pronounced improvements in the intervention group (*p* < 0.05). These findings indicate that structured exercise training effectively enhances immune function in AECOPD patients, potentially breaking the vicious cycle of inflammation-immunity imbalance.

Acute exacerbation of chronic obstructive pulmonary disease (AECOPD) often leads to skeletal muscle dysfunction and muscle atrophy. The involvement of respiratory muscles can exacerbate the progression of the disease and increase the burden of disease symptoms, thus forming a vicious pathological cycle (37). Our study demonstrated that graded exercise rehabilitation significantly improved multiple functional parameters in both groups, including reductions in mMRC and CAT scores, increased 6-min walk distance, and enhanced ADL scores (*p* < 0.05 versus baseline), with superior improvements observed in the intervention group compared to controls (*p* < 0.05). These findings indicate that structured exercise training effectively enhances both respiratory and locomotor function in AECOPD patients. The therapeutic mechanisms likely involve professionally supervised training targeting both respiratory mechanics (improving inspiratory/expiratory muscle strength and preventing fatigue) and peripheral muscle function. Through individualized prescription of exercise frequency, intensity, and duration, early initiation of rehabilitation promotes pulmonary function recovery, increases exercise tolerance, and alleviates dyspnea severity.

However, rehabilitation treatment should attach importance to the psychological state of patients. Studies by Rahi et al. reported that the incidence of anxiety and depression in patients with COPD was 30 to 50% (38). Chen Hua-ping et al.’s investigation and research on 154 COPD patients showed that 40.3% of COPD patients had varying degrees of anxiety and depression (39). Negative emotions such as anxiety and depression not only reduce patients’ compliance with treatment and rehabilitation exercises, but also cause abnormal excitement of the sympathetic nerve, resulting in physiological and pathological changes, and affecting the quality of life and disease prognosis of patients. The results of this study showed that the anxiety and depression scores of COPD patients in both groups before treatment were higher than the normal level. After treatment, the anxiety and depression scores of the intervention group were lower than those of the control group, and the difference was statistically significant (*p* < 0.05), suggesting that scientific and effective rehabilitation training can significantly improve the negative emotions such as anxiety and depression of COPD patients.

The results of this study indicate that the hospitalization time, non-invasive mechanical ventilation time, and the incidence of complications in the intervention group were significantly lower than those in the control group. This suggests that implementing graded exercise rehabilitation can reduce the hospitalization time, non-invasive mechanical ventilation time, and the occurrence of complications in patients with AECOPD. The intervention group received graded exercise rehabilitation, while the control group only received conventional exercise rehabilitation. Due to the lack of personalized plans, the physical functions of the control group declined, making it difficult to effectively improve and maintain lung function. The body was more prone to being invaded by pathogens or experiencing physiological dysfunction, increasing the risk of complications. For example, long-term lack of exercise leads to slowed gastrointestinal peristalsis, which may cause gastrointestinal distension; weak respiratory muscles are unable to effectively expel phlegm, resulting in phlegm accumulation in the airways, which may lead to difficulties in expectoration and other related complications.

This study has confirmed that graded exercise rehabilitation provides an effective personalized treatment plan for patients with acute exacerbation of chronic obstructive pulmonary disease (AECOPD), significantly improving immune function, inflammatory response, respiratory capacity, exercise endurance and disease burden, while reducing the duration of mechanical ventilation and hospitalization. These findings offer novel theoretical foundations and practical guidance for AECOPD rehabilitation with substantial clinical applicability. Future investigations should elucidate the precise mechanistic pathways of graded exercise rehabilitation and evaluate its synergistic effects when combined with other therapeutic modalities to develop more comprehensive and optimized rehabilitation strategies.

### Study limitations

This study has some limitations that need to be taken into account in future research: Firstly, the sample size is small and a single-center design is used, which may limit the statistical power and generalizability of the results. Moreover, due to potential selection bias in the recruitment of regional participants, the research results cannot be generalized to different medical environments. Secondly, although the two-week intervention period shows short-term therapeutic effects, it is unable to assess the long-term sustainability and prognostic impact because there is a lack of subsequent evaluation. Additionally, the study is a single-blind design (only the evaluators are blinded), which introduces potential execution bias because neither the participants nor the intervention providers are aware of the group allocation. Furthermore, potential confounding factors such as infection causes, comorbidities, and environmental factors (such as weather changes) were not adequately considered for their impact on the research results. Although efforts were made to maintain the relative balance of patients’ conditions and physical states during the study, these factors may still interfere and affect the accuracy of the conclusions. Future studies will take more rigorous measures to address this issue.

## Conclusion

Grade-based exercise rehabilitation has significantly improved the inflammatory levels, immune function and exercise capacity of patients with AECOPD through personalized plans. At the same time, it has shortened the hospital stay and reduced complications. The mechanism of its effect may be related to regulating the immune-inflammation balance, enhancing respiratory muscle function and providing psychological support. In the future, it is necessary to expand the sample size and extend the follow-up time to verify the long-term efficacy and explore the synergistic effect with other treatments. This study provides evidence-based evidence for the rehabilitation treatment of AECOPD patients and supports the inclusion of grade-based exercise rehabilitation in clinical practice.

## Data Availability

The original contributions presented in the study are included in the article/supplementary material, further inquiries can be directed to the corresponding author.
