# Correction: Effects of graded exercise rehabilitation on inflammatory factors and T-lymphocyte subsets in patients with acute exacerbation of chronic obstructive pulmonary disease: a randomized controlled trial

**DOI:** 10.3389/fmed.2026.1875577

**Published:** 2026-05-29

**Authors:** Yue Chen, Hong-Min Ran, Yan Wang, Dan-dan Fu, Na-na Yang, Chuan-li Cheng, Rong Liu, Lu-wen Luo, Ji-mei Luo, Li-na Ma, Hui Zeng

**Affiliations:** 1 The Second Affiliated Hospital of Zunyi Medical University, Zunyi, Guizhou, China; 2 Nursing School of Zunyi Medical University, Zunyi, Guizhou, China; 3 The Affiliated Hospital of Zunyi Medical University, Zunyi, Guizhou, China; 4 Department of Nursing, Zhejiang Cancer Hospital, Hangzhou, Zhejiang, China

**Keywords:** graded exercise rehabilitation, COPD exacerbation, immune function, inflammatory markers, T lymphocyte subsets

There was a mistake in Table 3 as published. The mMRC baseline scores were displayed as “4.22 ± 0.749” in the intervention group and “4.00 ± 0.734” in the control group. The corrected mMRC row in Table 3 appears below.

**Table 3:** Comparison of general data between the two groups.

Characteristic	Intervention group (*n* = 35)	Control group (*n* = 35)	Statistics	*p*-value
mMRC, score [mean (standard deviation)]	3.11 ± 0.83	2.86 ± 0.77	1.340	0.185

There was a mistake in Figure 4 as published. The mMRC values in Figure 4 were incorrectly displayed. The corrected complete Figure 4 appears below.

**Figure 4:**
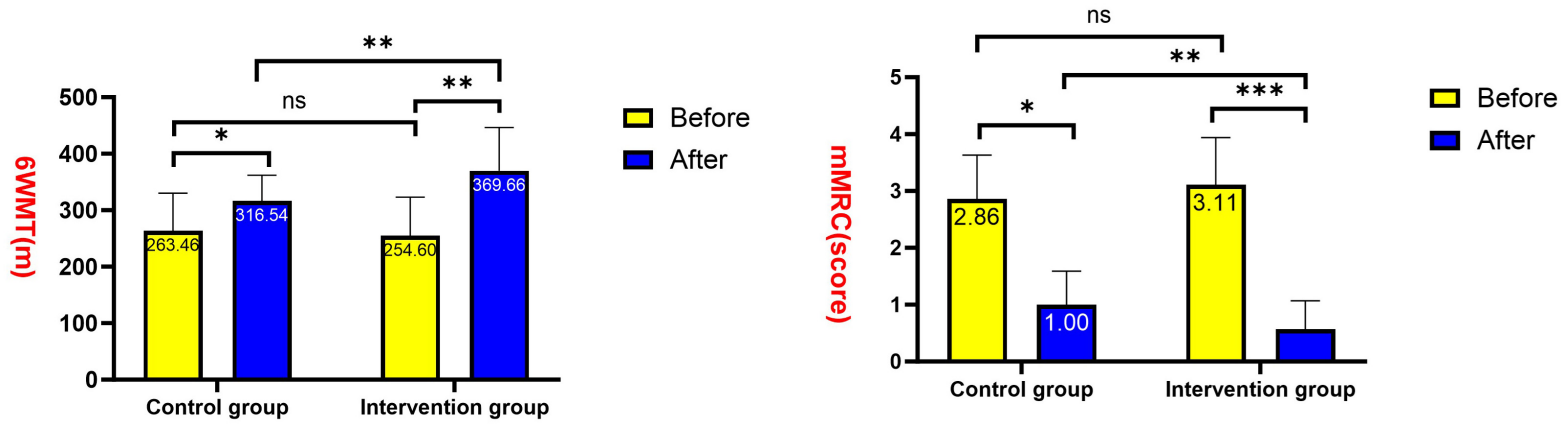
Comparison of 6MWT (meters) and mMRC (score). *p < 0.05, **p < 0.01, and ***p < 0.001.

In the **Abstract**, the intervention duration and assessment time points required clarification. The sentences:

“**Objectives:** To evaluate the effects of a 2-week graded exercise rehabilitation program on respiratory function, exercise capacity, inflammatory markers, and immune function in patients with acute exacerbation of chronic obstructive pulmonary disease (AECOPD).”

“The control group received conventional symptomatic treatment and exercise rehabilitation, while the intervention group underwent graded exercise rehabilitation according to the GOLD guidelines, twice a day, each session lasting 30–45 min, for a total duration of 2 weeks.”

“All evaluations were conducted 2 weeks before and after the rehabilitation treatment.” have been corrected to read:

“**Objectives:** To evaluate the effects of an in-hospital graded exercise rehabilitation program on respiratory function, exercise capacity, inflammatory markers, and immune function in patients with acute exacerbation of chronic obstructive pulmonary disease (AECOPD).”

“The control group received conventional symptomatic treatment and exercise rehabilitation, while the intervention group underwent graded exercise rehabilitation according to the GOLD guidelines, twice a day, each session lasting 30–45 min, during hospitalization until discharge.”

“All evaluations were conducted at baseline and at the end of the in-hospital rehabilitation intervention before discharge.”

In the **Materials and methods section**, subsection Interventions, the intervention duration was incorrectly described. The sentence:

“After a rigorous evaluation, if the patient's condition and vital signs are stable, exercise rehabilitation training will begin within 24 h after admission and will last for 2 weeks.”

has been corrected to read:

“After a rigorous evaluation, if the patient's condition and vital signs were stable, exercise rehabilitation training was initiated within 24 h after admission and continued during hospitalization until discharge.”

In the **Materials and methods section**, subsection Outcome measurement, Clinical outcomes, the unit for duration of non-invasive ventilation was incorrectly written. The sentence:

“Duration of non-invasive ventilation (total hours), Length of hospitalization (days), Incidence of complications (%).” has been corrected to read:

“Duration of non-invasive ventilation (days), length of hospitalization (days), incidence of complications (%).”

In the **Results section**, subsection The results of secondary observation indicators are as follows, the mMRC post-intervention values were incorrectly reported. The sentence:

“Post-intervention analysis revealed significantly better performance in the intervention group compared to controls for both 6-min walk test distance (369.66 ± 76.69 m vs. 316.54 ± 45.4 m, *p* = 0.001) and modified Medical Research Council (mMRC) dyspnea score (1.57 ± 0.5 vs. 2.00 ± 0.59, *p* = 0.002).” has been corrected to read:

“Post-intervention analysis revealed significantly better performance in the intervention group compared to controls for both 6-min walk test distance (369.66 ± 76.69 m vs. 316.54 ± 45.40 m, *p* = 0.001) and modified Medical Research Council (mMRC) dyspnea score (0.57 ± 0.50 vs. 1.00 ± 0.59, *p* = 0.002).”

The original version of this article has been updated.

